# Hormonal and genetic risk factors for breast cancer in a subset of the Karachi population

**DOI:** 10.1016/j.jtumed.2021.12.006

**Published:** 2022-02-01

**Authors:** Fouzia Shaikh, Mohiuddin Alamgir, Sehrish Ahmed

**Affiliations:** a Department of Pathology, Ziauddin University, Karachi, Pakistan; b Department of Pathology, Dow International Medical College, Dow University of Health Sciences, Ojha Campus, Karachi, Pakistan

**Keywords:** سرطان الثدي, النمط الجيني, مستقبلات الهرمون, تعدد اشكال طول القطعة المحدد, تعدد اشكال نيوكلوتيد وحيد, مستقبلات جين فيتامين د, Breast cancer, Genotype, Hormone receptors, RFLP, Single nucleotide polymorphisms, VDR gene

## Abstract

**Objective:**

Appraisement of vitamin D receptor (VDR) polymorphisms is thought to be crucial to detect and make approaches targeting groups at risk for breast cancer (BC). Moreover, an understanding of genetic susceptibility can allow us to foresee several risk factors. The objective of our research is to evaluate the T to C base shift within TaqI (rs731236) in exon 9 and the A to G transition within Bsm1 (rs1544410) in intron 8 of the VDR gene as risk factors among BC patients.

**Methods:**

The study involved 150 BC patients with a definite histological diagnosis. Controls were age-matched. DNA samples of Taq1 and Bsm1 were amplified according to the programmed protocol using a thermal cycler. The amplified PCR products were digested with Taq1 and Bsm1 restriction endonuclease enzymes. RFLP fragments were observed under UV light using 2% agarose gel and 0.5 ug/mL Ethidium bromide.

**Results:**

The highest number of BC patients (32.7%) were in the 36 to 45 age group. Ethnicity and parity were found to be statistically significant. TaqI polymorphisms showed the highest genotypic frequency for TC (Tt) at 49 (32.7%), and there were 18 patients (12.0%) and controls with high statistical significance (OD 3.6, CI 2–6.4) and a *p*-value < 0.0001. However, for the Bsm1 genotype, the A (B) allele may be linked with protection from BC in individuals with the AA (BB) genotype.

**Conclusion:**

A positive association was found between VDR genotypes and BC in a collective assay of Taq1 and BsmI. These results need further authentication in large cohort studies prior to applying these SNPs as promising BC markers in the Pakistani populace.

## الملخص

**أهداف البحث:**

يعتقد ان تقييم تعدد اشكال مستقبلات فيتامين د خطوة حاسمة لاكتشاف واتخاذ نهج من اجل استهداف مجموعة معرضة للخطر بسرطان الثدي. بالإضافة الى ذلك، فهم القابلية الوراثية يمكن ان يسمح لنا بالتنبؤ بالعديد من عوامل الخطر. يهدف هذا البحث الى تقييم التحول الأساسي لبعض الجينات التي تمت تجربتها لمستقبلات جين فيتامين د كعوامل خطورة لمرضى سرطان الثدي.

**طرق البحث:**

كان عدد المرضى مع التشخيص النسيجي المؤكد۱٥٠. تم مطابقة المجموعة الضابطة مع العمر. تم تضخيم عينات الحمض النووي للجينات وفقا لبروتوكول مبرمج باستخدام جهاز تدوير حراري. تم هضم تفاعل البلمرة المتسلسل المتضخم مع انزيمات نوكليازات الاقتطاع الداخلية للجينات. تمت ملاحظة تعدد اشكال طول القطعة المحدد تحت ضوء الأشعة فوق البنفسجية باستخدام ٢٪ هلام اجاروز و ٠٫٥ بروميد الايثيديوم٠

**النتائج:**

كان أغلبية مرضى سرطان الثدي (٧,۳٢٪) بين الفئة العمرية ۳٦ الى ٤٥. وجدنا ان العرق وتعدد الانجاب ذا دلالة إحصائية. اظهر تعدد اشكال الجين الأول اعلى تردد وراثي للحالات والمجموعات الضابطة مع دلالة إحصائية عالية. ومع ذلك ممكن للنمط الجيني الثاني، أ (ب) أليل ان يكون مرتبطا بالحماية من سرطان الثدي، عند الأفراد الذين يحملون النمط الجيني أأ ( ب ب).

**الاستنتاجات:**

هناك ارتباط إيجابي بين الانماط الجينية لمستقبلات فيتامين د مع سرطان الثدي في الفحص الجماعي بداخل الجينات التي تمت دراستها. تحتاج هذه النتائج لمزيد من المصداقية في دراسات مجموعات كبيرة قبل تطبيق تعدد اشكال النيوكلوتيد الوحيد كعلامات واعدة لسرطان الثدي لدى العامة في باكستان.

## Introduction

Breast cancer (BC) is the most frequently occurring carcinoma among females. It is the second most common cause of cancer mortality worldwide within the age range of 15–54.^1^ BC is the most prevalent and frequently diagnosed malignancy, with 119,710 cases, according to an assessment of the 5-year incidence rate, and 34,038 newly diagnosed patients as well as 16,232 fatalities in the year 2012 in Pakistan.^2^ BC is the most prevalent carcinoma not only in Asia but also around the globe. In the Western world, due to superior screening programs, BC is mainly detected in its early stages, allowing for timely treatment. However, in our region, due to poor health care facilities and lack of awareness regarding screening and self-examination protocols, this malignancy is the primary cause of most regional cancer-related deaths. According to Globocan, approximately 2.3 million new cases were reported in the year 2020, representing 11.7% of all cancer cases. BC accounts for 1 in 4 cancer cases and 1 in 6 cancer deaths, ranking first for incidence in the vast majority of countries (159 of 185 countries). The highest mortality rates are found in Melanesia, Western Africa, Micronesia/Polynesia, and the Caribbean (where Barbados has the world's highest BC mortality rate). Extensive research has revealed that 20%–30% of new BC cases are associated with factors that can be characterised as hormonal or nonhormonal including inherent causes like age, sex, race, family history, benign breast disease, and genetic makeup. Genetic polymorphisms may play a pivotal role in carcinogenesis.^3^ Each of these creates a separate parameter and remains constant during an individual's life. The most significant ones are age (i.e. being 40 years and older), history of breast disease, BC in a close blood relative, early age at menarche, birth of first child after age 35, menopausal age, and European population. The likelihood of BC increases by 3% with each 1-year delay in menopause. Every year of delay in menarche or each additional birth decreases the risk of BC by 5% or 10%, respectively.^4^^,^^5^ High dietary consumption of treated red meat has been studied for links to DNA damage, gene mutation, chromosomal damage, and epigenetic effects, genotoxicity, oxidative stress stimulation, and modified cell proliferation, cell death, and nutrient support.^6^ Familial BC is a cluster of BC within a family. However, in approximately 75%–80% of females, no risk factor is found, and most cases occur sporadically.^7^ Around 20% of patients have a family history of BC, and 5%–10% are hereditary BCs, showing germline mutation within BRCA1, BRCA2, TP53, or PTEN.^8^^,^^9^ It is essential to validate these BC susceptibility genes and assess the magnitude of the related risks.^10^ It is most likely that nonhormonal risk factors, along with common allelic variants of various genes, contribute to the development of BC. Low-penetrance susceptibility alleles, also known as ‘modifier genes’, have definitive alleles that are linked with modified risk or disease predisposition.^11^ One such low penetrance gene is the vitamin D receptor (VDR) gene, which has many allelic variants.^12^

The majority of the recognised functions of vitamin D are facilitated via the attachment of the active type of vitamin D to the VDR, which, being a ligand binding inducible transcriptional factor, is expressed in the majority of tissues, including in normal breasts and most BCs. The effective function of VDR genotypes BsmI, TaqI, and Fok1 is still obscure.^13^ Common genetic polymorphisms of VDR generate variants that differ among racial groups.^14^ The VDR gene holds several genetic polymorphisms at exon 2, 9, intron 8, and the 3′UTR region; researchers often investigate VDR-FokI (rs2228570), VDR-BsmI (rs1544410), and VDR-TaqI (rs731236).

Vitamin D and its new generation analogue are recognised as preventative and therapeutic mediators for BC.^15^ The appraisement of VDR polymorphisms is thought to be crucial to detect and make policies for targeting at risk groups. The objective of the current research was to evaluate whether the T to C base shift within TaqI (rs731236) in exon 9 and the A to G transition within Bsm1 (rs1544410) in intron 8 of the VDR gene were risk factors in a samples of BC patients.

## Materials and Methods

### Recruitment of study participants

One hundred and fifty BC patients with a definite diagnosis participated in this study. Patients were selected from the breast clinic at the Jinnah Postgraduate Medical Centre and the Baitul Sukoon cancer hospital in Karachi. Controls were age-matched with the patients’ age distribution by 5-year age groups; patients had no blood relative with a history of any malignancy (*n* = 150). Clinicopathological data regarding the tumour characteristics of the primary BC were obtained from medical records (tumour types, grades, ER, PR, Her2, and TNM classification). All materials used in the study were procured by Thermo-Fisher Scientific and Promega (USA).

(i) Cases of invasive BC proved by tissue biopsy and (ii) patients who consented to provide tissue and blood samples were included. Participants without a family history of BC and those who were not willing to give consent or had any other malignancy (in their history) were excluded.

### Sample collection

Whole blood samples were drawn before surgery and any chemotherapy treatment. Five ml non-fasting blood samples were collected in EDTA tubes (ethylenediaminetetraacetic acid). For genomic DNA extraction, peripheral blood was used by Chelex® 100 Resin (www.bio-rad.com).

Spectrophotometric measurement of DNA concentrations was performed on Nanodrop 2000 (Thermo Scientific, USA). DNA extraction samples were stored at −80 °C before analysis.

### Primer selection and genotyping

DNA samples of Taq1 and Bsm1 were amplified according to the programmed protocol using a thermal cycler (BIOER XP). Optimised pairs of primer were used for the VDR gene (Taq1 and BsmI), as previously reported.^16^^,^^17^ Amplification was performed using polymerase chain reaction (PCR) in 25 μL final reaction volume, mainly using Go Taq Green Master Mix, as per the manufacturer's protocols (Promega, USA). Amplification products for both Taq1 and Bsm1 were separated by size on a 2% agarose gel electrophoresis stained with ethidium bromide and visualised under a UV light Gel Doc system. The size of the digested products was established using 50 bp and 100 bp molecular markers on the DNA ladder. The amplified products for Taq1 and Bsm1 were 501 bp and 461 bp, respectively; fragments contained the genotype variant site. Single nucleotide polymorphisms TaqI and Bsm1 of the VDR gene were perceived via restriction enzyme digestion employing TaqI and Bsm1 endonuclease digestion (Thermo Scientific). RFLP fragments were separated using 2% agarose gel. Primer sequence and reaction conditions are shown in Table 1.

**Table 1:** PCR cycling conditions.

VDR/Taq1 (rs731236) (Torok R, 2013)^16^	Exon 9 (T/C)	5′-cagagcatggacagggagcaa-3′ 5′-cacttcgagcacaaggggcgttagc-3′ PCR program: x35:95 °C 60 s, 63 °C- 60 s 72 °C- 60 s Uncut PCR product: 501 bp Restriction enzyme: Incubation Temperature: Taq1, 65 °C Alleles: T:501, Allele C: 295 + 206 (bp)
VDR/BsmI (rs1544410) (Bagheri et al., 2012)^17^	Intron 8 (A/G)	5′-ggcaacctgaagggagacgta-3′ 5′-ctctttggacctcatcaccgac-3′ PCR program: x35: 95 °C 60 s, 67 °C 60 s, 72 °C 60 s undigested PCR product: 461 (bp) Restriction enzymes: Incubation temperature: BsmI, 37 °C Alleles: Allele A: 461, Allele G: 258 + 203 (bp)

SNP type and site, sequence of primer pair used for PCR amplification, undigested PCR products, restriction enzymes, incubation temperature, and allele size (bp) are summarised in Table 1.

## Results

### Questionnaire

**Figure 1:**
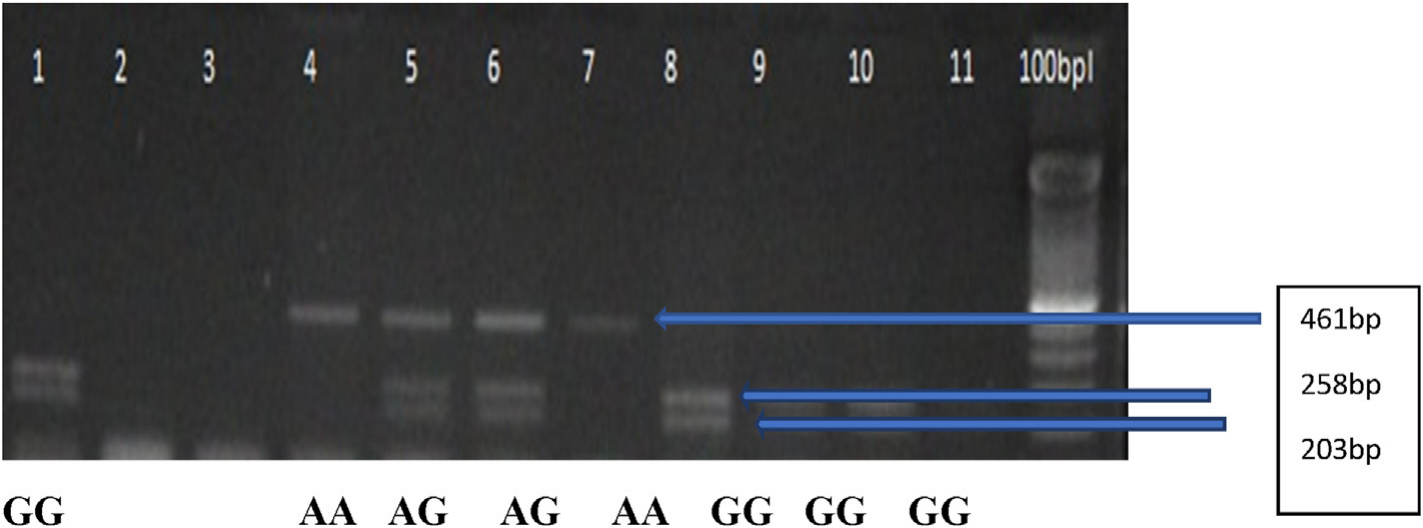
RFLP analysis of Bsm1 gene polymorphism. Lanes (1, 8, 9, 10) GG genotype. Lanes (4, 7) AA genotype. Lanes (5, 6) AG genotype. Lane (12): 100 bp DNA ladder.

**Figure 2:**
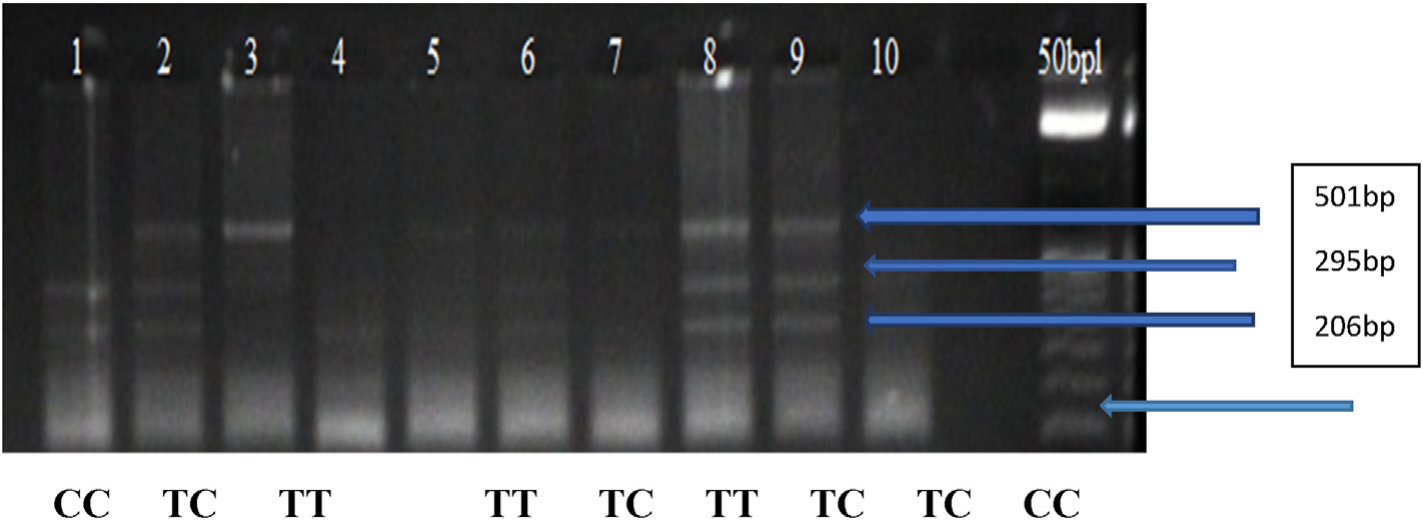
RFLP analysis of Taq1 gene polymorphism. Lanes (2, 6, 8, 9) TC genotype. Lane (1, 10) CC genotype. Lanes (3, 5, 7) TT genotype. Lane (12): 50 bp DNA ladder.

History concerning age, ethnicity, age at menarche, age at menopause, number of children, and account of breastfeeding was collected. To ascertain the outcomes of these variables, a binomial logistic regression was done on the probability that participants may have BC. These are the estimated multinomial logistic regression coefficients for the model. Two (parity and ethnicity) out of six predictor variables were found to be statistically significant (Table 2). The parity range for cases and controls was 0–>9, with a mean of 3 for cases and a mean of 1 for controls (see Figure 1, Figure 2).

**Table 2:** Logistic regression predicting likelihood of breast cancer placed on age, ethnicity, age of menarche, pre- and postmenopausal status, parity, and history of breastfeeding.

	B	S.E.	Wald	d	p-value	95% CI of Odds ratio	
						Lower	Upper
Age	−.265	.201	1.743	1	.187	.517	1.137
Ethnicity	.357	.090	15.765	1	.000	1.198	1.705
Menarche	.135	.143	.880	1	.348	.864	1.515
History of menopause	.791	.450	3.088	1	.079	.913	5.331
Parity	1.062	.201	27.858	1	.000	1.950	4.291
History of breastfeeding	−.207	.346	.359	1	.549	.413	1.601
Constant	−3.583	1.933	3.435	1	.064		

*p*-value ˂ 0.05 was considered statistically significant.

### Statistical analysis

Data were evaluated using IBM SPSS Statistics for Windows, Version 21.0. Armonk, NY: IBM Corp. Chi-square tests were applied for evaluating the association between Bsm1 and Taq1 genotype polymorphism and clinicopathological parameters. Binary logistic regression was applied between predictors (risk factors) and outcome variable. A *p*-value less than 0.05 was considered statistically significant.

A binomial logistic regression model was applied to determine the influence of age, ethnicity, age at menarche, history of menopause, history of breastfeeding, and parity to determine the possibility of participants having BC. The logistic regression model was statistically significant, χ^2^ (7) = 115, p=0.001). Among the ethnic groups, Urdu speaking patients had 1.4 times higher odds of breast carcinoma. Increased parity was associated with 3 times higher odds of exhibiting BC than having lesser parity.

The highest number of the BC patients (32.7%) were in the 36 to 45 age group. There were four patients who were below age 25 and 9 participants who were above age 65 years. A total of 55.3% were postmenopausal women. Parity status showed a maximum of 4–6 children (32%), whereas 49 (32.7%) controls had 1–3 children. More than 60.0% of the BC patients had a left side unilateral tumour, 50.7% had Grade 2, and 42.0% had a stage 2 tumour. The BsmI and Taq1 genotypes were correlated with histopathological tumour characteristics. A statistically significant association was not detected for any of the studied tumour parameters.

In our research, immunohistochemical analysis revealed that most of the BC cases were ER+ 91(60.7%) and PR+ 77 (51.3%), with more HER2/neu negative, 92 (61.3%).

The VDR BSM1 and Taq1 polymorphisms in our patients and control groups are depicted in Table 3, Table 4.

**Table 3:** Genotypic and allelic frequencies of Bsm1 polymorphism in breast cancer patients and healthy controls.

Bsm1 genotype	Breast cancer patients n-150 (%)	Healthy controls n-150 (%)	95% CI of Odds R	*p*-value
AA(BB)	40 (26.7)	61 (40.7)	0.53 (0.32–0.8)	0.001
AG(Bb)	37 (24.7)	51 (34)	0.6 (0.4–1.1)	0.007
GG (bb)	28 (18.7)	13 (8.7)	2.4 (1.2–4.9)	0.001
**Alleles**				
A(B)	77 (51.3)	112 (74.7)	0.4 (0.21–0.58)	0.001
G(b)	28 (18.7)	13 (8.7)	2.4 (1.2–4.9)	0.011

*p*-value < 0.05 was considered statistically significant.

**Table 4:** Genotypic and allelic frequencies of TAQ1 polymorphism in breast cancer patients and healthy controls.

Taq1 genotype	Breast cancer Patients n = 150 (%)	Healthy controls n = 150 (%)	Odds R (95% CI)	*p*-Value
CC (tt)	12 (8)	14 (9.3)	0.84 (0.3–1.9)	0.680
TC(Tt)	49 (32.7)	18 (12)	**3.6 (2–6.4)**	**0.001**
TT(TT)	23 (15.3)	15 (10)	1.6 (0.8–3.3)	0.170
**Alleles**				
C(t)	12 (8)	14 (9.3)	0.84 (0.3–1.9)	0.680
T(T)	72 (48)	33 (22)	**3.3 (2–5.4)**	**0.001**

*p*-value < 0.05 was considered statistically significant.

Bsm1 (A/G) polymorphisms located at the 3’ end of the VDR gene have the following genotypes: BB, Bb, and bb. The presence of a given restriction site was designated by lower case (b), while absence was designated by upper case (B).^18^ Regarding VDR polymorphism BsmI (rs154440), in both cases and controls, the genotype AA (BB) is statistically significant, with frequencies of 40 (26.7%), 61 (40.7%), and (OD 0.53, CI 0.32–0.8), and a *p*-value < 0.01. Similarly, allele A (B) is highly significant, with a *p*-value < 0.0001. The A (B) allele may be linked with reduced BC risk in individuals with the AA (BB) genotype. However, Taq1 (rs731236) polymorphism analysis shows that CT (Tt) heterozygous is statistically significant, with frequencies of 49 (32.7%), 18 (12%), and (OD 3.6, CI 2–6.4), and a *p*-value < 0.0001, as a result of the TC genotype and the T allele being considered risk factors for BC.

## Discussion

BC is a malignant disease. Its etiopathogeneses may comprise many genetic and ecological factors, along with several molecular signalling pathways. Age is the most important risk factor, along with gender. Race is a significant inherent factor increasing the incidence of BC.^19^ Ethnic variants and genetic disparities have been studied amongst populations, as no two individuals are genetically the same. In any given population, multiple variants of a single gene can exist. Ethnic discrepancies are also noted in VDR polymorphisms.^18^^,^^20^ Our study specifies the potential role of ethnic variances, together with other factors like living conditions and dietary practices that may contribute simultaneously. Although there has been advancement in the diagnosis and treatment of BC, ethnic discrepancies have continued for many years. Karachi is a cosmopolitan city comprising multi-linguistic people not only from Pakistan but also immigrants from the rest of the world. Nevertheless, our research is restricted to catchment areas near two oncology hospitals. Therefore, ethnic variance was justified. The majority of cases and controls belonged to the Urdu speaking ethnic group (53.3% and 36%), in accordance with other studies.^21^, ^22^, ^23^ The ethnic groups were differentiated from each other by multiple factors. These included socioeconomic status, geographical variations, lifestyle, and genetic makeup.^24^ A study conducted on BC in Baluchistan showed that the Pashtun ethnic group was highly affected by BC, with ages ranging from 41 to 50.^25^ Thus, ethnicity diversity might be recognised in future as a BC risk factor in our setup. In our region, early marriages are common, along with young age at the time of first childbirth, multiparity, and breastfeeding all children for an extended period. However, we found that cancer patients breastfed their children more often in contrast to controls, that is, 64% and 37.3%, respectively. In our society, women usually have more children than women in Western countries. This could be the reason for the low prevalence of BC in our population compared with the West. On the contrary, it was also reported that multiparity appeared to increase the threat of ER/PR/HER2 negative BC.^26^ However, the study^27^ also revealed that parity increases the risk of hormone receptor-negative BC, whereas breastfeeding lowers the risk or lessens the unfavourable effect of parity. This is inconsistent with the current research; we had more hormone positive and HER2/neu negative tumours. Various researchers have reported the controversial issues of parity and breastfeeding in the literature, predominantly the duration of breastfeeding and the number of parities. Certain researchers had revealed that single parity can decrease BC risk.^28^ Furthermore, Dall et al.^4^ suggested that parity in younger pregnancies could decrease the risk of BC. Hence, the soaring incidence of BC in Pakistan has yet to be elucidated. It cannot be deduced that breastfeeding alone can decrease the risk of BC because of the involvement of other confounders. These include parity, adiposity, and anovulation.^29^

In the current study, statistically significant correlation was not observed between ER, PR, and Her 2 Neu expression and clinicopathological parameters comprising tumour size, type, grade, lymph nodes, and the presence of distant metastases, as indicated in the results of previous studies.^30^^,^^31^ Currently, there is a scarcity of data on knowledge of low-penetrance genetic variants with oncogenesis of breast carcinoma. SNPs were found to be highly prevalent throughout the genome. Furthermore, high unpredictability makes these useful genetic markers beneficial for disease vulnerability.^32^ SNPs of the VDR gene were studied in two areas of this gene: an SNP in exon 9 (rs731236), known as TaqI RFLP, and an SNP at intron 8 called Bsm1 (rs1544410). In this population-based case–control study, we detected BC association with selected vitamin D-related gene polymorphisms, mainly Bsm1 (rs154440). The current data revealed that in individuals with the AA (BB) genotype, the A (B) allele can be associated with some degree of protection from BC. Similar findings were reported by Shahbazi et al. and Hashemi et al.^33^^,^^34^ regarding BsmI GG (bb) or even the AG (Bb) genotype. GG (bb) and G (b) were the second most common genotype and allele in the current research, with percentages of 18.7% and 8.7%, respectively, an OR of 2.4 (1.2–4.9 CI), and a *p*-value < 0.01. This made it a potential risk factor of BC. This finding is similar to those of Rashid et al. and Buyru et al.^35^^,^^36^ However, studies on Chinese and Pakistani populations did not report any link between Bsm1 VDR polymorphism and risk of BC incidence.^18^^,^^37^ In contrast to our findings, Utterlinden et al.^20^ reported the low incidence of the B allele of Bsm1 in the Asian population (7%) as compared to Caucasians (42%) and the African populace (36%).

For TaqI polymorphisms, our samples revealed that TC (Tt) had the highest genotypic frequency at 49 (32.7%) for cases and 18 (12%) for controls, with strong statistical significance (OD 3.6, CI 2–6.4) and a *p*-value < 0.0001. These results indicate that this genotype is a robust source for BC development. In keeping with our study, several studies have shown the frequent involvement of the heterozygous genotype TC (Tt) as genotype 71 (55%).^38^^,^^39^^,^^36^ As indicated in the literature, it can be deduced that the T allele is a ‘threat allele’, whereas the t allele can be a ‘defensive allele’.^40^ Overall, Mozaffarizadeh et al. produced the same results as ours, with Taq1 polymorphism displaying a significant association with BC risk. VDR Taq1 RFLP appears to be linked with BC development. We also found increased prevalence of the Tt genotype in BC patients compared with the healthy controls.

Certain findings of the current research can postulate the probability of females acquiring breast carcinoma. The research can also contribute to monitoring for new-found breast tumours, relapse, or distant metastasis, and further evaluating whether ‘at-risk’ genotypes are related to risk and disease progression.

## Conclusion

There appears to be a positive association between the studied VDR genotypes and BC in a collective assay of Taq1 and BsmI. Thus, VDR may be identified as essential for BC prevention. These results require further authentication in large cohort studies before the SNPs are applied as promising BC markers in the Pakistani populace. There was a limitation in our study that we were unable to acquire family history of controls.

We have demonstrated a positive association between VDR genotypes and BC in a collective assay of Taq1 and BsmI. However, further studies are needed for validation. We recommend incorporating multiple techniques such as western blotting for its validation.

## Source of funding

This research did not receive any specific grant from funding agencies in the public, commercial, or not-for-profit sectors.

## Conflict of interest

The authors have no conflict of interest to declare.

## Ethical approval

The study was conducted in agreement with the Declaration of Helsinki, as amended in 2013, for research relating to human subjects. The study protocol was initially assessed and later endorsed by the Ethics Review Committee at Ziauddin University (Reference No: 0450612SPath), and all participants signed written informed consent before interviews and blood collection.

## Authors contributions

This work was carried out through collaboration among all authors. FS and SA were involved in the conception of the idea and study design. MA performed data collection and bench work. FS supervised the project. SA and FS wrote the protocol of procedures and finalised the manuscript. SA performed the statistical analysis. FS and MA managed the literature searches. All authors have critically reviewed and approved the final draft and are responsible for the content and similarity index of the manuscript.
